# Comparison of Sexual Dysfunction Using the Female Sexual Function Index following Surgical Treatments for Uterine Fibroids

**DOI:** 10.1155/2012/368136

**Published:** 2012-08-23

**Authors:** Allison Ryann Louie, Jennifer Alice Armstrong, Laura Katherine Findeiss, Scott Craig Goodwin

**Affiliations:** Department of Radiological Sciences, UCI School of Medicine, Irvine, CA 92697, USA

## Abstract

Uterine fibroids are a common problem in women. Statistics showing 20–50% of fibroids produce symptoms and consequently patients seek surgical intervention to improve their quality of life. Treatments for fibroids are typically successful in controlling the fibroid disease, yet sexual function following invasive surgical treatments for fibroids can be jeopardized. The Sexual Function Index (FSFI) is a valid instrument producing quantifiable reproducible results. In this paper three case reports are evaluated by the FSFI and compared between the following treatment groups: hysterectomy, myomectomy, and uterine embolization. Our goal is to illustrate how each of these treatment outcomes can result in sexual dysfunction and therefore decreased quality of life. Effects of invasive fibroid treatments on sexual functioning would be helpful in guiding patient's ultimate decisions regarding treatment.

## 1. Introduction

Uterine fibroids are a common problem in the population, many women experience symptoms and some seek surgical intervention to improve their quality of life. Unfortunately, this is not always the outcome. Some patients have a decrease in quality of life due to sexual dysfunction, which is an unexpected adverse event of treatment. Treatments for fibroids are typically successful, yet some women experience an even worse outcome due to adverse effects of the very surgery meant to improve their quality of life. An epidemiologic study published in 2003 found that 80% of African American women and about 70% of Caucasians had evidence of fibroids on ultrasound [1]. In this population, it is estimated that 20–50% of fibroids produce symptoms including menorrhagia, dysmenorrhea, pelvic pain and pressure, dyspareunia, and/or urinary frequency and urgency [2]. Severe symptomatic fibroids warrant surgical treatment, the standard being a hysterectomy or in patients desiring fertility myomectomy.

There is controversy in the medical literature regarding quality of life with regard to sexual function following surgical treatments for fibroid disease [3–6]. The controversy lies in the subjectivity of the topic, making it difficult to assess the baseline prevalence of sexual dysfunction in women with fibroids [7, 8]. To overcome this challenge the Female Sexual Function Index (FSFI) was established [9, 10]. This test has been established as a valid instrument producing quantifiable reproducible results. In this study, patient's sexual function, evaluated by the FSFI, is described for inpatients in the following treatment groups: hysterectomy, myomectomy, and uterine embolization. Our goal is to illustrate how each of these treatment outcomes affects sexual dysfunction and therefore quality of life.

## 2. Female Sexual Function

There are different theories regarding female sexual function. According to Masters and Johnson, the female sexual response consists of four successive stages: excitement, plateau, orgasmic, and resolution phases [11]. Kaplan proposed the aspect of “desire” and condensed the cycle into just three phases, removing the aspect of resolution [12]. The Kaplan model is widely accepted today but might not fully take into account nonbiologic factors and relationship context [13–15]. 

### 2.1. Anatomy and Physiology of Female Sexual Function

The pelvic autonomic nerves supply the blood vessels of the vaginal wall, which originate mostly from the inferior hypogastric plexus (IHP). The IHP is essential for lubrication and sensation of the internal genitalia [16, 17]. The sensation of the external genitalia is linked to the pudendal nerve; this somatosensory nerve supplies the labia and clitoris, and reaches the external genitalia through a canal within the pelvic floor [18]. A number of studies related to hysterectomy and female sexual functioning have led researchers to conclude that disruption of the autonomic nerve supply leads to specific sexual disturbances. Disruption of the sympathetic nerve supply will lead to impaired lubrication and altered sensation of the female internal genital organs, and damage to the parasympathetic nerve supply will lead to impairment of vasocongestion [19, 20].

The hormonal milieu also plays an important role in female sexual functioning. Estrogen levels are related to the health of the vaginal wall and to lubrication, while androgens play an important role in sexual desire [21–24].

According to some research, there are two different types of female orgasm: internal and external. Some women describe the internal orgasm to occur when the pressure of the penis presses against the cervix. It has been suggested that the removal of the cervix can eliminate internal orgasm [25]. The external orgasm involves the clitoris, which is believed to produce sexual enjoyment for the majority of women [24]. External orgasm capability is reliant on the pudendal nerve, it supplies sensation to the external genitalia, that is, the labia and the clitoris [16]. The pudendal nerve can be compromised in hysterectomy and contribute to sexual dysfunction.

## 3. Sexual Dysfunction following Invasive ****Therapy for Uterine Fibroid Disease

### 3.1. Hysterectomy

#### 3.1.1. Case Report

The patient was a 52-year-old G1P1 female. She had menorrhagia secondary to five intracavitary submucosal fibroids. She also had a complex ovarian cyst on the right. She underwent a total abdominal hysterectomy and a right salpingo oophorectomy. She experienced postsurgical menopause after the hysterectomy associated with low testosterone levels. She experienced decreased sexual desire and difficulty in experiencing orgasm. She was treated with testosterone cream (Andro-Feme (Lawley Pharmaceuticals, Perth, Australia) and tibolone (Naari AG, Oberwil, Switzerland) which was then changed to an estradiol patch (Estradot, Novartis International AG, Basel, Switzerland). The FSFI questionnaire was administered. She reported scores for three time points. These included the time at which her sexual function was at its: (1) best prior to surgery- (32.2); (2) worst after surgery-(9.6); (3) best after hormonal therapy (17.5). Her scores are shown in Table 1. 

The results following hysterectomy can be both negative and positive. Positive outcomes include cessation of abnormal uterine bleeding, reduction in pelvic pain, and a decrease in depression and anxiety [26]. Generally, women display satisfaction with this procedure because their predisposing condition(s) are ultimately removed [27]. However, studies have shown that a significant proportion of women will develop new symptoms after hysterectomy, which include depression, fatigue, urinary incontinence, constipation, early ovarian failure, and sexual dysfunction [28, 29]. Many concerns have been raised regarding the appropriateness of this procedure for benign conditions, especially since it has an overall complication rate of 17–23% [30]. 

#### 3.1.2. Sexual Dysfunction Associated with Hysterectomy

The literature regarding sexual outcomes after hysterectomy is often conflicting and there remains debate as to what symptoms will be alleviated or improved after hysterectomy [5, 28, 31–33]. Some studies report that many women will experience sexual improvement due to the extirpation of a diseased uterus [5, 28, 29, 34] and that sexual desire and orgasm are likely to remain the same or become enhanced following surgery [35, 36]. Other investigators have estimated anywhere between 4% [37] and 40% [38] of women will report decreases in sexual desire after hysterectomy and an estimated 8% [39] to 25% [40] of women will experience decreased orgasmic ability.

The performance of a hysterectomy places a patient at risk of damage to the pelvic plexus [17]. With a more radical the surgery, the patient has a greater risk of nerve damage. Radical hysterectomy for cervical cancer causes greater nerve damage because the procedure involves the removal of the uterus and cervix as well as its lateral anterior and posterior ligaments [17]. These ligaments serve as pathways for the pelvic autonomic nerves; therefore, disruption of these nerves seems unavoidable during radical hysterectomy [17]. However, in 2003 new techniques were developed in radical hysterectomy to help identify and preserve the autonomic nerves, in hopes of preventing postoperative bowel, bladder, and sexual dysfunction [5, 41].

In relation to the cervix and sexual dysfunction, one hypothesis claims that removing the cervix can cause shortening and narrowing of the vaginal vault, which can result in severe dyspareunia and altered sensations during coitus. Formation of scar tissue in the vaginal cuff can also be a reason for dyspareunia [42]. It has also been suggested that the removal of the cervix can eliminate the experience of an internal orgasm [23].

Women who undergo oophorectomy experience a marked decrease in certain hormones which can cause symptoms that are typically seen in postmenopausal women, including sexual dysfunction. Oophorectomy patients experience a physiological decrease in estrogen and androgen levels, which results in reduced vaginal lubrication and dyspareunia [18]. An androgen deficiency is believed to be a cause of reduced libido and reduced sexual arousability [18–21].

The contributing factors that lead up to sexual dysfunction are multivariate. Although many investigators report hysterectomy does alter, to some proportion, the physiology of the female sexual response, there are still many researchers and studies that say otherwise. Moreover, the literature that addresses the costs and benefits of hysterectomy primarily focuses on overall efficacy and safety rather than on the patients who have particular adverse events such as sexual dysfunction.

### 3.2. Myomectomy

#### 3.2.1. Case Report

The patient was a 39-year-old G0P0 who underwent a myomectomy for the removal of 13 fibroids. Prior to myomectomy, she had dysmenorrhea, menorrhagia, dyspareunia, and bulk symptoms. Her postoperative course was complicated by an infection, yet 8 weeks post-op her symptoms were much improved. After four months, her dyspareunia returned and she was found to have three small fibroids. Four months later, she underwent laparoscopic surgery that revealed multiple adhesions. The adhesions were removed and the patients dyspareunia resolved post-op. The FSFI questionnaire was administered. She reported scores for five time points. These included the time at which her sexual function was at its: (1) best prior to surgery (33); (2) worst just prior to surgery (20); (3) best after first surgery (32.2); (4) worst after first surgery (24.9); (5) after second surgery (32.6). Her detailed scores are shown in Table 2.

Myomectomy is an invasive surgical procedure that removes fibroids and conserves the uterus; therefore, it has become the standard procedure for patients who wish to preserve fertility [43]. It can be performed hysteroscopically, laparoscopically, or via laparotomy, depending on the size, location, and number of fibroids, and the experience of the surgeon [44].

Myomectomy can only be performed on patients with fibroids of a certain number, size, and position [43]. Although the procedure has shown to relieve symptomatic fibroids, its main disadvantage is a 4%–30% recurrence rate [45].

Myomectomy is associated with multiple adverse outcomes such as blood loss, a risk of emergency conversion to hysterectomy, disfigurement of the uterine cavity, adhesions, and increased risk of uterine rupture in the case of pregnancy [46].

#### 3.2.2. Sexual Dysfunction Associated with Myomectomy

A literature review did not reveal any adverse effects of sexual dysfunction following myomectomy, with the exception of those related to adhesions, which are common in any abdominal surgical procedure [46]. Approximately 40% of chronic pelvic pain cases are directly related to adhesions [46, 47]. Pain from adhesions is probably related to their effect on adjacent structures. For example, it is known that adhesions around the ovaries can be associated with pain probably secondary to obstruction of the tubes and traction on the ovary [48, 49].

More studies are needed in order to investigate the relationship is between postmyomectomy adhesions and sexual functioning. Although assurance of a successful medical outcome is important, patients' postoperative quality of life is also essential.

### 3.3. UAE

#### 3.3.1. Case Report

The patient was a 40-year-old G4P3SAB1. On ultrasound she had a 20 cm uterus. Her symptoms included dysmenorrhea and menorrhagia. She underwent uterine artery embolization (UAE) and her symptoms improved. The FSFI was administered for three time points. These included the time at which her sexual function was at its: (1) best prior to UAE (34.5); (2) worst after UAE (19.8); (3) best after hormonal therapy (34.5). Her detailed scores are shown in Table 3.

A successful UAE procedure results in the resolution of presenting symptoms, such as menorrhagia, pain, urinary frequency, or constipation, without additional therapy [50]. UAE provides many benefits such as preservation of the uterus, avoidance of general anesthesia, a lower complications rate, and a substantially shorter recovery period [51]. UAE and fertility are currently under investigation and therefore not recommended for women who are trying to conceive [51]. 

Fibroid recurrence with UAE is thought to be 5% per year and is largely attributed to incompletely infarcted fibroids [52–54]. Overall, UAE has been reported to be an effective minimally invasive therapeutic alternative with high patient satisfaction rates [55–58].

#### 3.3.2. Sexual Dysfunction Associated with UAE

UAE has rarely been shown to have sexual complications. When it does occur, it is due to unintended embolization of the cervicovaginal branches of the uterine artery, which may lead to impairment in achieving orgasm and lubrication [6, 59]. Yet, many studies have displayed positive results concerning sexual functioning after UAE. A minority of patients report sexual dysfunction compared to the other fibroid treatments. For the welfare of future UAE patients, Lai et al. have suggested that objective questionnaires, complex hormonal evaluations, and complete sexual histories be completed to evaluate and stratify the risk of sexual dysfunction following UAE [59].

## 4. Conclusion

The postoperative sexual function and subsequent quality of life is an important part of a successful procedure. Unfortunately, the topic of sexual function is insufficiently discussed in the literature.

Patients undergo procedures with the expectations to alleviate symptoms and improve their quality of life. The expectations of the outcome should be discussed along with the risks and benefits of each procedure.

For the welfare of future hysterectomy, myomectomy, and UAE patients, additional attention should be paid to sexual function. Clinicians have proposed that in addition to the patient and physician discussion, there should be objective questionnaires and hormonal evaluations [59].

These case reports and the literature illustrate the possibility of sexual dysfunction after invasive fibroid procedures for fibroid disease. Further studies on the effects of invasive fibroid treatments on sexual functioning would be helpful in guiding patient's ultimate decisions regarding treatment.

## Figures and Tables

**Table 1:** Hysterectomy patient FSFI.

Domain	Score		
time point	1	2	3
Desire	3.9	1.2	3
Arousal	5.7	1.2	1.8
Lubrication	6.0	1.2	4.7
Orgasm	6.0	1.2	2
Satisfaction	5.6	1.2	1.6
Pain	6.0	3.6	4.4
Full scale score	**32.2**	**9.6**	**17.5**

(i) Since being on Estrodot—she has claimed that the hormones have increased libido but do not help arousal and orgasm—which have become very difficult and weak and rarely happen.

(ii) Currently, hysterectomy PT can only experience clitoral orgasm after surgery. She stated that only 10–20% of the time she experiences orgasm from coital stimulation.

(iii) Because of abdominal hysterectomy by mid-line incision, she has experienced a changed perception of herself; she feels that her body does not look its age and feels that she has lost part of identity.

**Table 2:** Myomectomy patient FSFI.

Domain	Score				
time point	1	2	3	4	5
Desire	4.8	4.2	4.8	4.2	4.8
Arousal	5.4	4.2	5.4	4.5	5.4
Lubrication	6.0	4.8	6.0	4.8	6
Orgasm	6.0	3.6	6.0	4.4	6
Satisfaction	5.6	2	5.2	4	5.6
Pain	5.2	1.2	4.8	3	4.8
Full scale score	**33**	**20**	**32.2**	**24.9**	**32.6**

**Table 3:** UAE patient FSFI.

Domain	Score		
time point	1	2	3
Desire	4.8	6.0	6.0
Arousal	5.7	1.2	5.7
Lubrication	6.0	4.2	6.0
Orgasm	6.0	1.2	4.8
Satisfaction	6.0	1.2	6.0
Pain	6.0	6.0	6.0
Full scale score	**34.5**	**19.8**	**34.5**
